# Wild Oats

**Published:** 1899-11-25

**Authors:** 


### WILD OATS.

Among the social phenomena of the present century one of the most striking has been the growth of great cities and the absorption by them of an ever-increasing proportion of the population.

Coincidently with this extension of city life has grown up another characteristic of the century which is of no small importance, namely, a growing prevalence of diseases affecting the nervous system, including, as we now can hardly any longer doubt, a vast increase in the number of the insane. For this the town-bred man, as is his wont, frames for himself excuses which fan his vanity. He would say that this decadence of the nervous system is but another proof that the town intellect is of "finer fibre" and is more "highly strung" than that type which he is pleased to call bucolic, and that by dint of its very activity it wears out the sooner.

To some extent this may be true, and no one can doubt that the stress, the competition, and the anxiety of modern life have much to do with that large prevalence of nervous diseases which seems to be one of its inevitable accompaniments. But other causes are in operation. However much it may please the town man nowadays to plume himself on the possession of a feather-edged nervous system, much as it pleased our forefathers to believe that all their ailments arose from gout, we must go further if we want the truth. The tendency of modern pathology is to drive back the etiology of nerve disease to the devitalising effects upon the nervous tissue of certain poisons, which are conveniently classed as toxins; or to disturbances of nutrition arising from diseases of the blood-vessels or connective tissue, which are themselves also often due to similar toxins; and among these toxic influences the virus of syphilis stands supreme. Thus we see how true is the old phrase, "*Mens sana in corpore sano.*" In pride of intellect, we say that the gross body is ruled and kept in subjection by something ethereal which we call mind. But in truth the mind is but the very humble servant of the body, and is dependent on it even for its nutrition; and this brings us round to the relation between nervous disease and civilisation. It is not merely because the mind which works in towns is sensitive and highly-strung that it breaks down, but because it is fed by a town-bred body, and one which is too often poisoned by town-bred disease.

Opinion has long been drifting steadily towards the conclusion that such well-marked affections of the nervous system as tabes and general paralysis of the insane, although not syphilitic in the ordinary sense, and not capable of cure by the remedies which are found effectual in that disease, are still the results of injuries inflicted upon the nervous system by that infection. Speaking recently of the relation between syphilis and general paralysis of the insane, Dr. Mott, Pathologist to the Asylums of the London County Council, pointed out that syphilis was "the most important factor" in the causation of that disease. The same may be said of tabes; indeed, Dr. Buzzard, speaking on this subject at the meeting of the Pathological Society last Tuesday, insisted on the identity of the change in these two diseases, and said that syphilis was the predominant and far the most important factor in their production. It is difficult to avoid the conclusion that in regard to many of the less understood diseases of the nervous system a similar relationship holds good. When we are told, as we are told, that such a disease as general paralysis occurs especially among those exposed to the stress and worry and excitement of a highly developed civilisation, and when we are told how it has increased as towns have grown, we cannot shut our eyes to the other concomitants of life in great cities. Civilisation, town life, and syphilis seem indeed to run together. We know how largely this disease is responsible for the premature ageing which is so characteristic a feature of the town-living men; we know how extensively it affects the blood vessels, and thus leads to morbid states of nerve structure, which, although not syphilitic, are still the results of syphilis, and it is year by year becoming clearer that outside and beyond the range even of such lesions as these, its poison has an injurious influence upon nervous tissues (analogous perhaps to that exercised by the poison of influenza), by which their vitality is reduced, and they are predisposed to fail when subjected to stress.

We can have no doubt, and we always maintain, that much of the ill health of town dwellers is due to the surroundings amid which they live. The overcrowding, the dirt, the lack of light, all tend to lower their vitality and to leave them peculiarly at the mercy of disease. But it is not entirely from these causes that the dweller in big cities deteriorates, and that his nervous system tends to give way. The life he leads is probably quite as important as the surroundings amid which he leads it, for all modern knowledge concerning the wide distribution of the after effects of syphilis goes to show that the sowing of wild oats is not so simple an affair as some imagine.

